# Risky, protective, and screening health behaviours among childhood cancer survivors: A cross-sectional survey in Australia and New Zealand

**DOI:** 10.1007/s11764-025-01858-6

**Published:** 2025-07-02

**Authors:** Lauren Ha, Christina Signorelli, Jordana K. McLoone, Claire E. Wakefield, Joseph E. Alchin, Venkatesha Venkatesha, Richard J. Cohn, Joanna E. Fardell

**Affiliations:** 1 Behavioural Sciences Unit, Discipline of Paediatrics & Child Health, School of Clinical Medicine, UNSW Medicine and Health, UNSW Sydney, Randwick, Australia; 2 Kids Cancer Centre, Sydney Children’s Hospital, Randwick, Australia; 3 Division of Quality of Life and Pediatric Palliative Care, Department of Pediatrics, Stanford Medicine Children’s Health, Palo Alto, CA USA; 4 Northern Sydney Local Health District Executive, Royal North Shore Hospital, St Leonards, NSW Australia; 5 Western Sydney Youth Cancer Service, Westmead Hospital, Westmead, Australia

**Keywords:** Childhood cancer survivors, Health behaviour, Alcohol, Smoking, Physical activity, Sun protection, Screening, Dental

## Abstract

**Purpose:**

Childhood cancer survivors are at risk of late effects. Engaging in health behaviours may reduce this risk. We aimed to investigate engagement in risky (alcohol consumption, smoking), protective (sun protection, physical activity, dental hygiene), and screening (cervical screening, skin examinations) behaviours between survivors and an age- and sex-matched control group.

**Methods:**

We conducted a cross-sectional survey in childhood cancer survivors (aged 16 + years) who were 5 + years post-diagnosis, proxy report of parents of survivors (aged < 16 years) and age- and sex-matched non-cancer controls. We compared survivor responses to the Children’s Oncology Group Long-Term Follow-Up Guidelines.

**Results:**

612 survivors (median age = 19, range = 7–61; 49% male) and 423 controls (median = 21, range = 0–45; 51.5% male) were included in our analysis. Survivors were more likely to smoke less (*p* < .001), floss less (*p* < .05), use sunscreen (*p* < .001), wear protective clothing (*p* < .001), stay in the shade less (*p* < .01), and engage in skin examinations (*p* < .001), compared to controls. Most survivors met alcohol (72%), smoking (93%) and sunscreen (66%) guidelines. Fewer survivors met guidelines for physical activity (31%), flossing (4%), wearing a hat for sun protection (40%), skin examinations (40%) and cervical screening (43%). Survivors who were older age, female sex and had higher income were associated with meeting all health behaviour guidelines.

**Conclusions:**

Many survivors do not meet protective and screening behaviour recommended guidelines.

**Implications for Cancer Survivors:**

Engagement in multiple positive health behaviours is critical for this population who are at increased risk of developing long-term health issues.

**Supplementary Information:**

The online version contains supplementary material available at https://doi.org/10.1007/s11764-025-01858-6.

## Background

The survival rate for childhood cancer now exceeds 85% in Australia and most other high-income countries [1–3], leading to a growing population of cancer survivors [4]. While the increased survival rates are encouraging, the risk for secondary cancers and chronic health conditions [5, 6], early mortality [7], and poor quality of life[8] are major challenges for this population. Due to exposure to toxic cancer therapies at a young age, a high proportion of childhood cancer survivors experience an array of physical, psychosocial, and cognitive late effects, many of which do not become clinically apparent until decades later [9–12]. Therefore, maximising quality of life and reducing late effects after cancer treatment is crucial for promoting survivors’ long-term health.

Engaging in health protective behaviours such as regular exercise and a healthy diet, plays an important role in reducing the risk of chronic conditions such as obesity, second cancers and cardiovascular disease among cancer survivors [13, 14]. Yet research suggests that childhood cancer survivors seldom meet recommended physical activity or dietary guidelines [15, 16]. Additional lifestyle factors such as tobacco smoking, alcohol consumption, sun protection, dental hygiene, and screening behaviours are known to influence the risk of disease among the general population [17–21]. For cancer survivors, adhering to multiple health behaviour recommendations is fundamental to maintaining overall health and wellbeing, given survivors’ increased risk for cancer recurrence, second cancers and late effects. For example, patients treated with cardiopulmonary toxic agents (such as anthracyclines) or radiation therapy are strongly advised to abstain from tobacco use as it may lead to cardiac toxicity and serious pulmonary complications [22, 23]. Other therapeutic exposures such as cyclophosphamide used as chemotherapy, especially prior to secondary dentition and radiation therapy to the head and neck, have been associated with dental anomalies [24, 25]. Regular dental examinations and good dental health practices are crucial for this population. Engaging in cancer surveillance and screening, such as mammography and cervical testing, is another important health behaviour with the potential for early detection and more effective management, given survivors’ increased risk of recurrent or secondary cancers [26, 27].

In the general population, smoking, alcohol binge drinking, physical inactivity, and consuming an unhealthy diet frequently co-occur and are correlated [28]. Assessing and then addressing multiple health behaviours concurrently may maximise health benefits [29, 30], reduce financial burden, and improve confidence or self-efficacy to modify other health behaviours [31]. While various studies have investigated childhood cancer survivors’ adherence to single health behaviour recommendations [32, 33], or compared health behaviour adherence to a control group of people without cancer [34–36], evidence is limited regarding the simultaneous practice of healthy lifestyle behaviours, as associated with cancer-specific guidelines, with comparisons to an age- and sex-matched group. In this context, we use the following definitions: i) ‘risky’ health behaviours are those that increase the risk of negative impacts of behaviours such as alcohol consumption and tobacco use after cancer and/or cancer treatment, ii) ‘protective’ health behaviours are those that reduce the severity or likelihood of negative late effects of cancer and/or cancer treatments, including sun protection, physical activity, and dental hygiene, and iii) ‘screening’ behaviours are those that may detect early signs of cancer including cervical screening and skin examinations.

Therefore, the objectives of this study were to:

1. Describe and compare engagement in risky, protective, and screening behaviours between childhood cancer survivors and an age- and sex-matched non-cancer control group.
2. Assess engagement in risky, protective, and screening behaviours in relation to recommended cancer-specific guidelines among survivors.
3. Identify demographic and clinical factors associated with engagement in protective, risky, and screening behaviours in survivors.

## Methods

### Data source

Data in this study were collected as part of a larger study: the Australian & New Zealand Children’s Haematology/Oncology Group (ANZCHOG) Survivorship Study [37]. The study was approved by the relevant ethics committees for all participating hospitals (HREC/12/POWH/345) and the University of New South Wales (HC15773).

## Participants

We recruited childhood cancer survivors from 11 Australian and New Zealand paediatric hospitals to complete a questionnaire (2013–2016). We included childhood cancer survivors who were diagnosed with any cancer before the age of 16 years, were 5 + years post-diagnosis at the time of study participation, had completed active cancer treatment at one of the 11 participating hospitals, and were proficient in English. For eligible survivors who were below 16 years of age, we invited parents to complete the survey on behalf of their child. Parents also completed surveys for survivors with cognitive, mental health, or language difficulties that would have prevented them from completing the survey themselves. Therefore, one questionnaire was completed per family (either by the survivor or a parent). There were no additional exclusion criteria, thus all survivors who met the inclusion criteria were retained in the study. For the control group, we recruited Australian adults without cancer via an online survey community (Pureprofile Pty Ltd). Eligible control participants were healthy individuals aged 18 + years who were proficient in English. We excluded control participants who had any health condition, any history of cancer or who were a parent or guardian of a child with a history of cancer.

## Procedure

As detailed elsewhere [37], we identified survivors through medical records of their treating hospital. We mailed questionnaires to survivors with an invitation letter, consent form, and reply-paid envelope. For eligible survivors aged between 16–18 years, we addressed invitation letters to both survivors and their parents, with the decision of participation made by the family. For our control group, we advertised the study online via Pureprofile Pty Ltd. To ‘opt in’ to the study, control participants completed an online consent form and questionnaire and subsequently were reimbursed $5 AUD for completion.

## Measures

Participants self-reported clinical and demographic details. The questionnaire took participants approximately 30 min to complete. Demographic data included age, sex, education attainment, employment status, marital status (married/de facto or not married/de facto), place of residence, and annual household income before tax (≤ $60,000 AUD or > $60,000 AUD). Clinical data included primary cancer diagnosis, age at diagnosis, treatment completion date, and treatment history (surgery, chemotherapy, radiotherapy, bone marrow transplant).

We assessed participants’ risky (alcohol consumption and tobacco smoking), protective (physical activity, sun protection, dental hygiene), and screening (skin examinations, cervical screening) behaviours. Health behaviour survey items were developed by a multidisciplinary expert panel comprising of a behavioural scientist, psychologist, oncologist, and specialist cancer nurse. These items were then reviewed and tested for clarity and relevance with two consumer representatives. The health behaviours included in the questionnaire were carefully selected based on prior research and known late effects experienced by childhood cancer survivors [38–40]. Items were designed to enable comparison with recommended guidelines ensuring contextual relevance and to facilitate interpretation of findings.

For alcohol consumption, we asked participants to record the number of standard drinks of alcohol they had consumed in the past two weeks. Participants were also asked to report their tobacco smoking status at the time of study participation (either ‘never smoked’, ‘ex-smoker’, ‘current smoker (daily)’, or ‘current smoker (less than 1 daily)’). To assess moderate-to-vigorous physical activity (MVPA) levels, we asked participants to indicate the time in minutes per day, ‘In the past two weeks, how much time did you spend doing moderate/intense exercise?’. To capture sun protection behaviours, we asked participants to report on their perceived level of engagement (‘never’, ‘sometimes’, ‘often’, or ‘always’) with five protective behaviours during periods when they were in the sun for more than 15 min between 10am and 4 pm. These included i) using SPF 30 + sunscreen, ii) applying sunscreen 15 min before going outside, iii) wearing a shirt with sleeves, iv) wearing a wide-brimmed hat, and v) staying in the shade or using an umbrella. We assessed dental hygiene by asking participants, ‘do you floss every day’ with response options ‘never’, ‘rarely’, ‘sometimes’, ‘often’, ‘always’. We asked all survivors about two screening behaviours (i.e., skin examinations and cervical screening) regardless of their risk factors and treatments received. For skin examinations, we asked participants, ‘In the last 24 months (2 years), how often have you had a doctor examine your moles and skin for changes?’ with the response options, ‘never’, ‘once’, ‘twice or more’. Similarly, we assessed cervical screening behaviours by asking participants, ‘In the last 24 months (2 years), how often have you had a pap smear test?’ with response options, ‘never’, ‘once’, ‘twice or more’, and ‘not applicable’.

We compared survivors’ risky, protective and screening behaviours to the Children’s Oncology Group (COG) ‘Long-Term Follow-Up Guidelines for Survivors of Childhood, Adolescent, and Young Adult Cancers Version 4.0’, the version in use at the time of the study (Table 1) [41]. For dental hygiene and sun protection behaviours, we categorised those who reported ‘often’ or ‘always’ as meeting the COG guidelines. For skin examinations, we categorised those who reported ‘once’ or ‘twice or more’, as meeting the COG guidelines. For cervical screening, we excluded all males and all survivors under the age of 21 years in accordance with cervical screening guidelines in place at the time of data collection, which recommended initiating screening at age 21. We categorised survivors as meeting the COG guidelines if they reported screening'once'or'twice or more,'in accordance with the recommendations at the time of data collection. Although cervical screening guidelines have since been updated to recommend screening at age 25 and every five years [42, 43], our analysis is based on the guidelines that were in place during the data collection period.

**Table 1:** Health behaviours compared to the Children’s Oncology Group (COG) Long-Term Follow-Up Guidelines for Survivors or Childhood, Adolescent, and Young Adult Cancers Version 4.0 [41]

Health behaviour	COG Guidelines
Alcohol consumption	For men, limit alcoholic drinks to < 2 standard drinks per day For women, limit to one standard drink per day
Tobacco smoking	No smoking
Physical activity	For adults (18 + years), engage in ≥ 150 min/week of moderate-to-vigorous physical activity For children and adolescents (< 18 years), engage in 60 min/day of moderate-to-vigorous physical activity
Sun protection	Always wear protective clothing or sunscreen when skin is exposed to the sun Wear sun protection factor (SPF) sunscreen of 15 or higher Avoid outdoor activities from 10am to 2 pm
Dental health	Floss at least once a day
Skin examinations	Individuals who have risk factors (e.g., radiation to any area, underwent haematopoietic cell transplant or ever had skin cancer or melanoma) to have a thorough skin examination at least once a year
Cervical screening	Cervical cancer screening should begin at age 21 years Women ages 21 to 29 should receive a pap test every 3 years Women ages 30 to 65 should receive a human papillomavirus (HPV), and pap test every 5 years, or pap test alone every 3 years (alternative)

COG: Children’s Oncology Group; min: minutes.

## Statistical analysis

We applied propensity score matching to minimise cofounding by age and sex for survivors and controls. Firstly, we estimated propensity scores for each participant using logistic regression. Participants were matched on propensity scores using a nearest-neighbour algorithm without replacements, with a 1:2 ratio between survivors and controls. We applied a calliper of 0.2 standard deviations of the logit of the propensity score to ensure the matches were sufficiently proximal to each other. After matching, we conducted balance diagnostics to confirm that the distribution of age and sex between survivors and controls were comparable, with standardised mean differences below 0.1. For Aim 1, we summarised the data using mean (SD) for continuous normally distributed data and median (interquartile range [IQR]) for ordinal or non-normal data. We used frequencies or percentages to present categorical variables. To compare the risky, protective, and screening behaviours between survivors and controls, we used independent samples t-test for normally distributed data or the Mann–Whitney U test for skewed data. We compared categorical variables using the Chi-square test or Fisher’s exact test to account for sparsely distributed data. For aim 2, We determined engagement in risky, protective, and screening health behaviours by a summative index scoring method, guided by Tollosa and colleagues [44]. One point was given for those who were adhering to each COG guideline and zero points for those who were not meeting each COG guideline. For Likert-scale responses, we scored ‘always’ and ‘often’ with one point and ‘sometimes’, ‘rarely’ and ‘never’ with zero points (Supplementary File S1). These individual health behaviour scores were then summed to produce a total score for each of the health behaviours (risky behaviour score = 0–2; protective behaviour score = 0–3; screening behaviour score = 0–2 for females and score = 0–1 for males). For Aim 3, we used ordinal logistic regression in a multivariable model to estimate the odds for meeting risky, protective, and screening health behaviour guidelines, adjusting for covariates (age, sex, place of residence, education status, employment status, marital status, household income). We also employed a general linear model to assess the association of variables with meeting all health behaviours collectively. Missing data were handled under the assumption that data were missing at random. Given the low extent of missing data (< 5%), all analyses were conducted using available cases without the application of imputation methods [45]. We performed our statistical analyses using RStudio[46] and IBM SPSS Statistics 28.0 (IBM Corp.) [47].

## Results

In total, 634 survivors (54% participation rate) and 510 controls (population without cancer) returned a completed questionnaire. After propensity score matching, our final matched dataset included 612 survivors and 423 controls. Survivors were median aged 19 (IQR 15–27) years and 49% were male. Controls were median aged 21 (IQR 13–29) years and 51.5% were male. Survivors were on average 15.8 (SD 7.5) years post cancer diagnosis. Most survivors were diagnosed with leukaemia (43.8%), followed by a solid tumour (42.4%) and lymphoma (12.2%). Table 2 summarises the clinical and sociodemographic characteristics of survivors and controls.

**Table 2:** Participant characteristics table for survivors and controls

**Outcome**	**Survivors (N = 612)**	**Controls (N = 423)**	**p value**
**Participant group, *n (%)***			
Self-report	430 (70.3%)	298 (70.4%)	
Parent-report	182 (29.7%)	125 (29.6%)	
**Age, years** ***median (IQR)***	19 (15–27)	21 (13–29)	0.52
*Range*	7–61	0–45	
**Sex,** ***n (% male)***	298 (48.7%)	218 (51.5%)	0.40
**Place of residence** ^**a**^ ***n (%)***			
Metropolitan	423 (76.6%)	341 (80.8%)	0.45
Regional or remote	129 (23.4%)	81 (19.2%)	
**Education** ^**b**^ ***n (%)***			
High school or apprenticeship	237 (39.4%)	119 (28.1%)	**< 0.001**
Further study including degrees and certificates	364 (60.6%)	304 (71.9%)	
**Employment** ^**c**^ ***n (%)***			
Employed	444 (73.9%)	298 (70.4%)	0.27
Not employed	158 (26.1%)	125 (29.6%)	
**Household income** ^**d**^ ***n (%)***			
≤ $AUD 60,000	248 (47.9%)	130 (37.7%)	**< 0.05**
> $AUD 60,000	270 (52.1%)	215 (62.3%)	
**Marital status** ^**e**^ ***n (%)***			
Married or defacto	290 (47.7%)	221 (52.2%)	0.17
Never married or defacto, single or widowed	318 (52.3%)	202 (47.8%)	
**Primary cancer diagnosis** ^**f**^ ***n (%)***			
Leukaemia	267 (43.8%)	**NA**	
Solid tumour	258 (42.4%)		
Lymphomas	74 (12.2%)		
Other	10 (1.6%)		
**Treatment received** ^**g**^ ***n (%)***			
Surgery	277 (47.7%)	**NA**	
Chemotherapy	575 (92.6%)		
Radiotherapy	248 (42.6%)		
Bone marrow/stem cell transplant	110 (19.6%)		
**Years since cancer diagnosis,** ***mean (SD)***	15.8 (7.5)	**NA**	
**Years since treatment completion,** ***mean (SD)***	13.7 (7.4)	**NA**	

**IQR**: Interquartile Range where Q1-Q3, **N**: Number of participants; **NA**: Not applicable; **SDs**: Standard deviation.

Mean, standard deviation, and range are reported for continuous variables, such as age.

Numbers and percentages may not add up due to missing values and rounding errors.

^*a*^ Regional defined as ARIA = inner regional, outer regional, rural or remote; Metro defined as ARIA = major city. Missing data for survivor ARIA (n = 60), missing data for control ARIA (n = 1).

^b^ Missing data for survivor education (n = 11).

^*c*^ Missing data for survivor employment status (n = 13).

^*d*^ Missing data for survivor (n = 82) and control (n = 78) household income, including the response option, ‘I’d prefer not to say’.

^*e*^ Missing data for survivor marital status (n = 6).

^*f*^ Missing data for survivor primary cancer diagnosis (n = 3).

^*g*^ Participants may have received more than one treatment.

### Health behaviour engagement

Table 3 summarises the descriptive statistics on health behaviours in survivors compared to controls. The median (IQR) for alcohol consumption per fortnight was 1 (0–3) standard drinks for survivors and 1 (0–3) standard drink for controls (W = 129.183, *p* = 0.374). We observed significant differences in smoking habits between survivors and controls (χ^2^ = 28.73, *p* < 0.001). Among survivors, 4.6% were ‘current daily smokers’, compared to 8.5% of controls. Similarly, 2.7% of survivors and 4.2% of controls reported ‘currently smoking less than daily’. The proportion of ‘ex-smokers’ was 7.7% among survivors and 15.1% among controls. The majority (85.0%) of survivors reported never having smoked, compared to 72.3% of controls.

**Table 3:** Descriptive statistics on risky, protective, and screening health behaviours among cancer survivors compared with controls

Outcome	Survivors (N = 612)	Controls (N = 423)	p-value
**Risky behaviours**			
**Alcohol consumption** ^a^			
*Median (IQR)*	1 (0–3)	1 (0–3)	0.374^+^
*Range*	0—56	0—50	
**Tobacco smoking** ^b^ *n (%)*			
Current smoker (daily)	28 (4.6%)	36 (8.5%)	**< 0.001**^**++**^
Current smoker (less than daily)	16 (2.7%)	17 (4.2%)	
Ex-smoker	47 (7.7%)	64 (15.1%)	
Never smoked	516 (85.0%)	306 (72.3%)	
**Protective behaviours**			
**Moderate-vigorous physical activity levels**, min/fortnight ^c^			
*Median (IQR)*	240 (90–480)	180 (60–480)	0.055^+^
*Range (minutes)*	0—4800	0—6000	
**Dental hygiene**, floss daily ^d^ *n (%)*			
Never	203 (33.5%)	138 (32.6%)	**0.017**^**++**^
Rarely	171 (28.1%)	116 (27.4%)	
Sometimes	155 (25.6%)	90 (21.3%)	
Often	56 (9.1%)	43 (10.2%)	
Always	23 (3.8%)	36 (8.5%)	
**Sun protection** *n (%)* ^e^			
**Used SPF30 + sunscreen**			
Never	30 (5.0%)	48 (11.3%)	**< 0.001**^++^
Rarely	174 (28.7%)	125 (29.6%)	
Often	249 (41.1%)	162 (38.3%)	
Always	153 (25.2%)	88 (20.8%)	
**Applied sunscreen 15 min before going outside**			
Never	85 (14.1%)	70 (16.5%)	0.651^++^
Rarely	232 (38.6%)	151 (35.7%)	
Often	207 (34.5%)	150 (35.5%)	
Always	77 (12.8%)	52 (12.3%)	
**Wore a shirt with sleeves**			
Never	23 (3.8%)	42 (10.0%)	**< 0.001**^++^
Rarely	113 (18.7%)	77 (18.2%)	
Often	258 (42.7%)	177 (41.8%)	
Always	210 (34.8%)	127 (30.0%)	
**Wore a wide-brimmed hat**			
Never	149 (24.7%)	119 (28.1%)	0.089^++^
Rarely	212 (35.1%)	145 (34.3%)	
Often	158 (26.1%)	120 (28.4%)	
Always	85 (14.1%)	39 (9.2%)	
**Stayed in shade or used umbrella**			
Never	90 (15.0%)	55 (13.0%)	**< 0.010**^**++**^
Rarely	235 (39.0%)	129 (30.5%)	
Often	225 (37.4%)	193 (45.6%)	
Always	52 (8.6%)	46 (10.9%)	
**Screening behaviours**			
**Skin examinations in past two years** ^f^, *n (%)*			
Never	365 (60.0%)	335 (79.2%)	**< 0.001**^**++**^
Once	139 (22.8%)	60 (14.2%)	
Twice or more	105 (17.2%)	28 (6.6%)	
**Cervical screening in past two years** ^g^, *n (%)*			
Never	131 (57.2%)	77 (57.9%)	0.273
Once	69 (30.1%)	46 (34.6%)	
Twice or more	29 (12.7%)	10 (7.5%)	

^+^*based on Mann–Whitney U test,* ^++^*Chi-square test*

^a^ Standard drinks per fortnight

^b^ Missing data for survivor smoking (n = 5). Reference value = never smoked

^c^ Missing data for survivor MVPA levels (n = 31)

^d^ Missing data for survivor dental hygiene (n = 4)

^e^ Missing data for survivor sun protection – using SPF30 + sunscreen status (n = 6); apply sunscreen 15 min before going outside (n = 11); wore shirt with sleeves (n = 8); wore wide brimmed hat (n = 8); stay in shade or use umbrella (n = 10)

^f^ Missing data for survivor skin examinations (n = 3)

^g^ Cervical screening only included for females aged 21 + years for survivors (n = 314) and non-cancer controls (n = 205). Missing data for survivors (n = 85) and controls (n = 72). The mean (SD) age for survivors was 28.9 (6.41) years and 29.2 (6.2) years for controls

Survivors and controls reported 240 min (IQR 90–480) and 180 min (IQR 60–480) of moderate to vigorous intensity physical activity per fortnight (W = 131,523, *p* = 0.056). Regarding dental hygiene, survivors reported a lower frequency of daily flossing (3.8% vs. 8.5%, χ^2^ = 11.94, *p* < 0.05). A higher proportion of survivors reported ‘always’ using sunscreen (25.2% vs 20.8%, χ^2^ = 16.09, *p* < 0.001), ‘always’ wearing a shirt with sleeves (34.8% vs 30.0%, χ^2^ = 16.51, *p* < 0.001), compared to controls. A lower proportion of survivors reported ‘always’ (8.6% vs. 10.9%) or ‘often’ (37.4% vs. 45.6%) staying in the shade or using an umbrella for sun protection compared to controls (χ^2^ = 11.22, *p* < 0.01). There were no significant differences between survivors and controls for applying sunscreen at least 15 min before going outside (χ^2^ = 1.64, *p* = 0.651) and wearing a wide-brimmed hat (χ^2^ = 6.49, *p* = 0.089).

In examining screening behaviours, 60.0% of survivors and 79.2% of controls reported not having undergone any skin examination in the past two years (χ^2^ = 45.17, *p* < 0.001). Some survivors (17.2%) and few controls (6.6%) reported two or more skin examinations. Most female participants (survivors and controls) reported not having engaged in cervical cancer screening in the past two years (57.2% vs. 57.9%). One-third of survivors and controls reported engaging in one (30.1% vs. 34.6%) and a small proportion reported having engaged in two or more cervical cancer screenings in the last two years (12.7% vs. 7.5%).

### Engagement in recommended guidelines

We assessed a total of two risky, seven protective, and two screening behaviour guidelines. Survivors reported meeting an average of 1.5 risky behaviour guidelines (SD 0.62, range 0–2), 1.4 protective behaviour guidelines (SD 1.4, range 0–7), and 0.3 screening behaviour guidelines (SD 0.5, range 0–2).

**Fig. 1:**
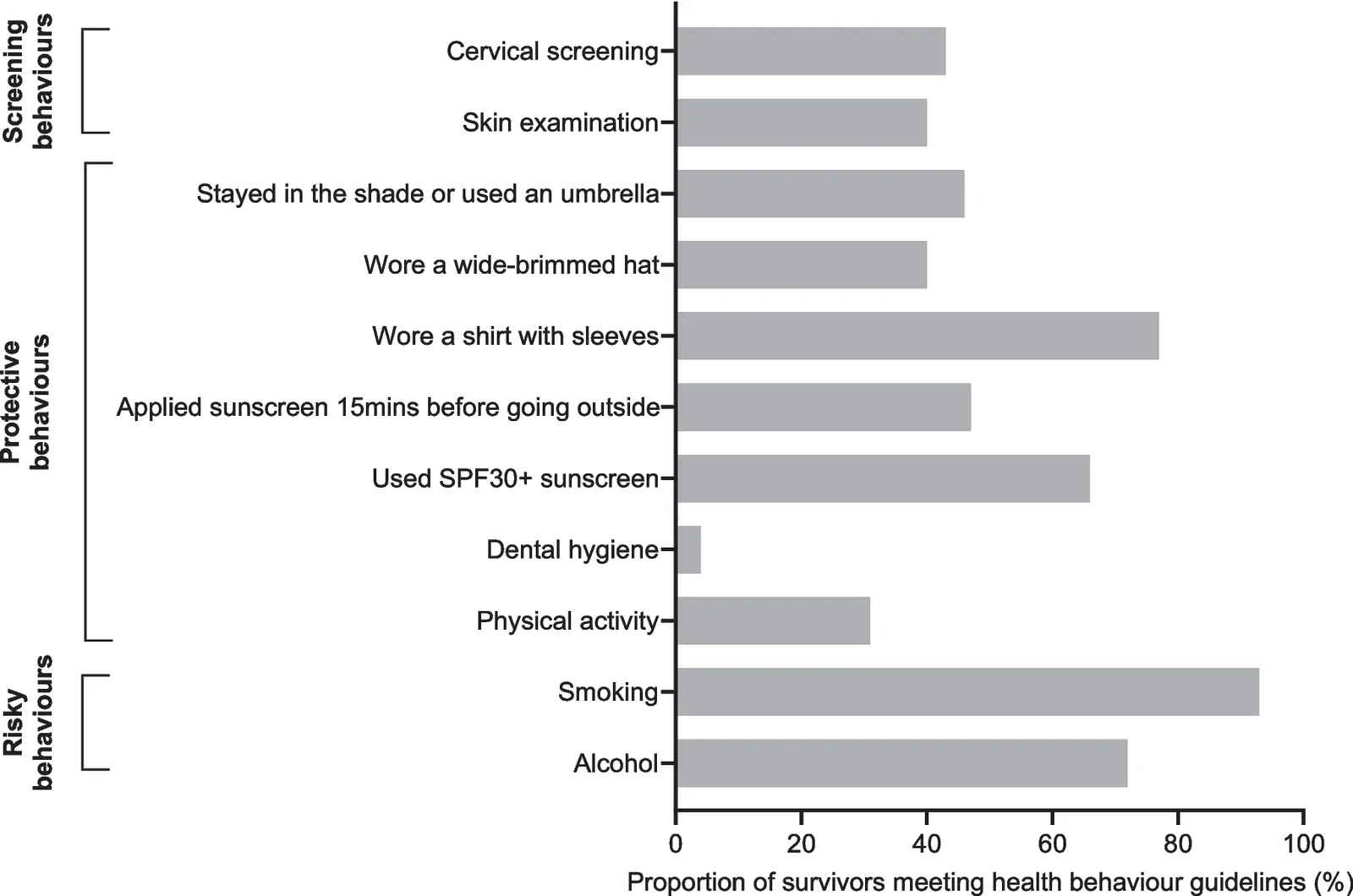
Proportion of survivors meeting the Children’s Oncology Group recommendations for risky, protective, and screening behaviours

Figure 1 displays the proportion of survivors meeting recommended health behaviour guidelines. Most survivors met alcohol (72%) and smoking (93%) guidelines. One-third of survivors (31%) met physical activity guidelines. Engaging in sun protective behaviour guidelines varied. The most common sun protective behaviour guideline met by survivors were wearing a shirt with sleeves (77%) and using SPF30 + sunscreen (66%). The guidelines of applying sunscreen at least 15 min before going outside and stayed in the shade or used an umbrella, were met by 47% and 46% of survivors, respectively. The least common sun protective guideline was wearing a hat (40%). Engagement in daily flossing guidelines were low among survivors (4%).

In examining screening behaviours, 40% of survivors met guidelines for skin examinations. Of those survivors who received radiotherapy, 124 (52%) had not had their skin examined in the last two years, 65 (28%) had their skin examined once, and 47 (20%) had their skin examined twice or more in the last two years. Of those survivors who received bone marrow/stem cell transplant, 52 (49%) never had their skin examined, 27 (25.5%) had their skin examined once, and 27 (25.5%) had their skin examined twice or more in the last two years. Among female survivors, 43% met cervical screening guidelines.

### Determinants of meeting health behaviour guidelines

We examined the determinants of survivors’ engagement in risky, protective, and screening behaviour guidelines through a multivariable model and identified several significant associations (Table 4). Older age was associated with higher odds of meeting risky (OR 1.12, 95% CI 1.09—1.16, *p* < 0.001) and screening behaviour guidelines (OR 1.07, 95% CI 1.05—1.10, *p* < 0.001). There were significant associations between sex and risky behaviours with females demonstrating higher odds of meeting risky (OR 1.59, 95% CI 1.08–2.34, *p* < 0.001) and screening behaviour guidelines (OR 3.12, 95% CI 2.02–4.80), *p* < 0.001). Higher income earners (OR 0.53, 95% CI 0.33–0.87, *p* = 0.011), currently employed individuals (OR 0.48, 95% CI 0.30–0.79, *p* = 0.004) and married individuals (OR 0.55, 95% CI 0.32–0.82, *p* = 0.005) were less likely to meet risky behaviour guidelines.

**Table 4:** Determinants of meeting risky, protective and screening behaviour guidelines—multivariable analyses based on ordinal logistic models

Multivariable analysis							
		**Risky**		**Protective**		**Screening**	
**Predictor**		**OR**^**+**^** (95%CI)**	**p**	**OR**^**+**^** (95%CI)**	**p**	**OR**^**+**^** (95%CI)**	**p**
Age, yrs		1.12 (1.09–1.16)	** < 0.001**	0.99 (0.95–1.03)	0.837	1.07 (1.05–1.10)	** < 0.001**
Sex	Male	1.0		1.0		1.0	
	Female	1.59 (1.08–2.34)	** < 0.001**	1.21 (0.62–2.38)	0.570	3.12 (2.02–4.80)	** < 0.001**
Residence	Metro	1.0		1.0		1.0	
	Rural	0.66 (0.43–1.04)	0.073	0.77 (0.36–1.65)	0.494	1.12 (0.70–1.78)	0.636
Education	High school only or apprentice	1.0		1.0		1.0	
	Post-school education	1.33 (0.87–2.02)	0.189	1.42 (0.69–2.93)	0.341	1.25 (0.80–1.95)	0.322
Income	< 60 K	1.0		1.0		1.0	
	> = 60 K	0.53 (0.33—0.87)	**0.011**	0.78 (0.34–1.79)	0.562	0.83 (0.51–1.34)	0.443
Employment	Unemployed	1.0		1.0		1.0	
	Employed	0.48 (0.30—0.79)	**0.004**	0.75 (0.32–1.74)	0.500	1.25 (0.76–2.08)	0.382
Marital status	Unmarried	1.0		1.0		1.0	
	Married	0.55 (0.32—0.82)	**0.005**	1.15 (0.52–2.56)	0.721	1.22 (0.78–1.90)	0.377

*OR = odds ratio, *^*+*^*based on ordinal logistic regression adjusting for age, sex, education,* income, marital status, employment status, and region of residence.

1.0 = reference category used in the estimation of OR.

Risky behaviours = alcohol consumption, smoking.

Protective behaviours = physical activity, dental hygiene, sun protection.

Screening behaviours = skin examinations, cervical screening.

Table 5 evaluates factors associated with meeting all health behaviour guidelines taken collectively in survivors. In the multivariable analysis, age exhibited a significant positive association with meeting guidelines, such that older survivors were more likely to meet all health guidelines (OR = 1.06, 95% CI 1.01–1.06, *p* < 0.001). Sex also emerged as a robust predictor, with females displaying significantly higher positive behaviours than males (OR = 1.71, 95% CI 1.09–2.09, *p *= 0.001). Higher income also positively correlated with meeting all health guidelines (OR = 0.61, 95% CI 0.51–1.08, *p* = 0.012). Marital status correlated with engagement, however, in the multivariable model, this association became non-significant (OR = 1.16, 95% CI 0.78–1.17, *p* = 0.465).

**Table 5:** Determinants of meeting all health behaviour guidelines taken collectively

		**Univariate**		**Multivariable**	
**Predictor**		**OR (95%CI)**	**p**	**OR (95%CI)**	**p**
Age, yrs		1.05 (1.03–1.07)	** < 0.001**	1.06 (1.01–1.06)	** < 0.001**
Sex	Male	1.0		1.0	
	Female	2.06 (1.55–2.75)	** < 0.001**	1.71 (1.09–2.09)	**0.001**
Residence	Metro	1.0		1.0	
	Rural	0.72 (0.51–1.04)	0.082	0.78 (0.57–1.22)	0.209
Education	High school only or apprentice	1.0		1.0	
	Post-school education	1.16 (0.87–1.55)	0.309	1.06 (0.91–1.85)	0.734
Income	< $60,000	1.0		1.0	
	> = $60,000	0.58 (0.42–0.78)	** < 0.001**	0.61 (0.51–1.08)	**0.012**
Employment	Unemployed	1.0		1.0	
	Employed	0.76 (0.55–1.05)	0.095	0.78 (0.68–1.50)	0.276
Marital status	Unmarried	1.0		1.0	
	Married	0.71 (0.54–0.95)	0.0**21**	1.16 (0.78–1.17)	0.465

Scoring based on Supplementary File 1. Higher score indicates higher engagement to health behaviour guidelines.

*OR* = *odds ratio,* ^+^*based on ordinal logistic regression adjusting for age, sex, education, income, marital status, employment status, and region of residence.* 1.0 = reference category used in the estimation of OR.

## Discussion

Childhood cancer survivors face an elevated risk of late effects and chronic conditions [6]. Not meeting health behaviour guidelines can further increase their vulnerability to adverse health outcomes [48]. Whilst previous research has documented health behaviour engagement among childhood cancer survivors, these behaviours were often considered in isolation. This study sought to compare risky (alcohol consumption and tobacco smoking), protective (physical activity levels, sun protection, dental health), and screening (skin examinations, cervical cancer screening) behaviours between survivors and an age- and sex-matched non-cancer control group, survivors’ health behaviour engagement compared to the recommended guidelines, and to identify factors associated with meeting health behaviour guidelines.

When compared to age- and sex-matched controls, our results indicate that childhood cancer survivors exhibit similar rates of alcohol consumption, physical activity and cervical cancer screening but lower rates of smoking and dental hygiene; and higher rates of having skin examinations. Sun protective behaviours varied between survivors and controls. Survivors reported greater rates of using SPF30 + sunscreen and wearing sun protective clothing yet reported lower rates of staying in the shade or using an umbrella. Our results align with previous literature, reporting survivors smoke less tobacco compared to controls [49, 50]. Although some literature supports our findings that survivors have similar patterns of alcohol consumption to their peers [50], there is mixed evidence [36, 51]. Differences in alcohol consumption rates among childhood cancer survivors across global studies may be explained by variations in demographics, cultural norms, cancer type and treatment, psychosocial factors, study design, and timing of assessment. Our physical activity results differ from previous studies, which have highlighted that survivors are less active than non-cancer controls [52, 53]. Our study sample may reflect a subgroup of survivors who were more physically active, more engaged in long-term follow up care, and therefore may be more exposed to health promotion messages and foster a greater awareness of healthy behaviours. As a result, survivors in this study may have been more motivated to adopt healthy behaviours, including physical activity. Previous studies further confirm that survivors exhibit similar or higher rates of sun protective behaviour, including wearing sunscreen and protective clothing [32, 54, 55].

Although survivors in this study overall reported more favourable health behaviour patterns than controls, they still fell short of the recommended guidelines for a population at increased risk of developing serious and life-threatening late effects [6]. In a study from the St. Jude Lifetime Cohort in 397 childhood cancer survivors aged 10–17 years, survivors reported low engagement with multiple health behaviour guidelines including physical activity, diet and sedentary/screen-based behaviours [56]. This low engagement was associated with poorer cardiometabolic health (i.e., higher resting heart rate) and physical performance (i.e., motor proficiency). Given the young age of this group of survivors, focusing on addressing multiple health behaviours will be critical for reducing the risk of long-term health outcomes, especially for young survivors as they establish lifelong habits.

Our data highlight that additional efforts are needed to improve physical activity, smoking and oral hygiene. Engaging in multiple behaviours that are detrimental to one’s health can have synergistic effects on health [57]. Among the adult cancer population, cancer outcomes are associated with multiple health behaviours. In breast cancer survivors, reduced cancer recurrence is associated with sufficient levels of physical activity [58], not smoking [59], and low or no alcohol consumption [60]. Increased risk of cancer recurrence is further associated with overweight and obesity [60]. Childhood cancer survivors therefore may benefit from routine long-term follow-up or risk-based health care to encourage healthy behaviours [61]. Future interventions targeting this population may also consider designing programs that address and promote multiple health behaviours concurrently, with considerations to involve peers and family members to reinforce engagement in healthy behaviours Further, it will be important to consider digital or telehealth models to improve accessibility for families living in urban, regional and remote areas.

For all health behaviours taken collectively, we demonstrated that older age, female sex and higher income were associated with better health behaviour engagement. These factors are consistent with previous research, indicating that older and female survivors may prioritise their health behaviours, potentially due to a greater awareness of risk [34, 36]. Previous research has also documented that families from lower socioeconomic status backgrounds may be more vulnerable to poorer health behaviour engagement [62]. Additional supports and attention are needed for younger, male survivors and individuals from lower socioeconomic areas. Future research may consider employing co-design methodologies to develop or adapt existing health behaviour interventions [63]. Engaging these sub-groups, who have lived experience, to collaborate with researchers and health professionals to collectively inform and enhance interventions may be valuable in ensuring interventions are effective [64]. Therefore, future interventions targeted at reducing smoking and alcohol consumption, increasing physical activity levels and improving dental hygiene may be effective if they address the factors impacting social disadvantage.

The strengths of this study include our large nationwide sample of participants living in Australia and New Zealand. We employed propensity score matching, to age- and sex-match our control group, minimising confounding variables that may impact their engagement in health behaviours. In addition, we assessed multiple health behaviours which provide a comprehensive understanding of the role of various lifestyle factors among childhood cancer survivors. This study also has some limitations that need to be considered. Whilst we had a relatively low participation rate in our study (54%), it is comparable to similar studies involving childhood cancer survivors [65]. We acknowledge the potential for response bias, whereby those who chose to participate may differ from non-respondents. The cross-sectional, self-report design of this study limits the conclusions that can be drawn from this research. Whilst our survey items were purpose-designed by an expert multidisciplinary team and reviewed by consumer representatives, the limited validated questionnaires may have introduced measurement bias. Not all participants may accurately recall their health behaviours and some may feel motivated to over- or under-report behaviours [66]. Parents may also not have been able to accurately assess their child’s health behaviours, such as physical activity during school hours, or may have been unaware of behaviours such as alcohol use and smoking. We did not assess vaping, which was not a common practice at the time of study recruitment. With a recent rise in uptake of nicotine products among young Australians, including dual users of smoking and vaping, new users who have never smoked, and those who have swapped smoking for vaping, vaping may be considered a new risky health behaviour that will be important to assess in the future [67]. We also did not assess other protective health behaviours including diet and tooth brushing. Regarding screening behaviours, we did not assess breast, bowel or cardiac screening which are important for the aging survivor population [68]. For cervical screening, we did not assess whether participants were sexually active and did not have data for those who were at high risk for cervical cancers (i.e., pelvic irradiation or treatments that increase the risk of cervical abnormalities), which may have influenced screening eligibility and engagement to guidelines. Further, our survey was designed at the time when the cervical screening recommendation was every two years. In this study, we compared participant responses to the COG Guidelines Version 4.0 [41], which were the recommendations at the time of data collection and engagement in cervical screening guidelines may differ in this sample if compared to the updated COG Guidelines [43]. Finally, all participants in this study were English-speaking and may not reflect the diversity of multicultural communities in Australia and New Zealand. Further investigation in health behaviour engagement among culturally and linguistically diverse populations are important for future interventions to lessen the inequity gap.

In conclusion, our findings indicate that overall, childhood cancer survivors exhibit lower rates of some risky behaviours and higher engagement in some protective and screening behaviours compared to age- and sex-matched controls, although their adherence to recommended guidelines remains lower than ideal for a high-risk population. While alcohol consumption, physical activity levels, and cervical screening behaviours were comparable between the two groups, given survivors’ already elevated risk of developing secondary cancers and chronic health conditions, targeted interventions are needed to encourage improvement in modifiable health behaviours. Future lifestyle interventions may consider targeting younger survivors, male sex, and those with lower household income.

## Supplementary Information

Below is the link to the electronic supplementary material.

Supplementary file1 (DOCX 22 KB)

## Data Availability

The data that support the findings of this study are not publicly available due to privacy or ethical restrictions, and the full dataset is not able to be released due to ethical restrictions. Requests may be made to the authors.
