# Supplementary material for: Risky, protective, and screening health behaviours among childhood cancer survivors: A cross-sectional survey in Australia and New Zealand
Source: J Cancer Surviv. 2025 Jul 2;20(Suppl 1):728–40. doi: 10.1007/s11764-025-01858-6 (PMC13676637; doi:10.1007/s11764-025-01858-6)
Supplement: Supplementary file 1 [file 11764_2025_1858_MOESM1_ESM.docx]

**Supplementary File S1**

|  | **Health behaviour variable** | **Scoring** | |
| --- | --- | --- | --- |
| **Risky behaviours*** | Alcohol consumption in the last fortnight | ≤20 SDs per fortnight | 1 |
|  |  | >20 SDs per fortnight | 0 |
|  | Smoking | Never smoked | 1 |
|  |  | Ex-smoker | 0 |
|  |  | Current smoker | 0 |
| **Protective behaviours**** | Physical activity levels | Active (≥60min/day or ≥150min/week) | 1 |
|  |  | Insufficiently active (0-59min or 0-149min) | 0 |
|  | Sun protection   - Use SPF30+ sunscreen - Apply sunscreen 15min before going outside - Wear a shirt with sleeves - Wear a wide-brimmed hat - Stay in the shade or use an umbrella | ‘Always’ or ‘often’ | 1 |
|  |  | ‘Sometimes’ or ‘never’ | 0 |
|  | Oral hygiene (flossing) | ‘Always’ or ‘often’ | 1 |
|  |  | ‘Sometimes’ or ‘rarely’ or ‘never’ | 0 |
| **Preventive behaviours***** | Skin examinations in the last two years | Twice or more | 1 |
|  |  | Once | 0 |
|  |  | Never | 0 |
|  | Cervical screening in the last two years | Twice or more | 1 |
|  |  | Once | 1 |
|  |  | Never | 0 |

* Maximum score for risky behaviours = 2

** Maximum score for protective behaviours = 3

*** Maximum score for preventive behaviours = 1 (male) and 2 (female)
